# Breakage of the dense structure of coal precursors increases the plateau capacity of hard carbon for sodium storage†

**DOI:** 10.1039/d4sc06549b

**Published:** 2024-12-02

**Authors:** Wen-Yu Qian, Xin-Yang Zhou, Xin-Yao Liu, Meng-Yuan Su, Kai-Yang Zhang, Xing-Long Wu

**Affiliations:** a MOE Key Laboratory for UV Light-Emitting Materials and Technology, Northeast Normal University Changchun Jilin 130024 China xinglong@nenu.edu.cn; b Faculty of Chemistry, Northeast Normal University Changchun Jilin 130024 China

## Abstract

Hard carbon is considered the most commercially viable anode material for sodium ion batteries due to its excellent sodium storage properties. However, the production cost of hard carbon is high, so optimizing the electrochemical performance of coal-derived hard carbon is adopted. However, due to the dense structure of coal, it is difficult to prepare closed pores inside the coal-derived hard carbon, which is not conducive to increasing capacity. Therefore, we propose Zn_2_(OH)_2_CO_3_ assisted ball milling pretreatment followed by carbonization to generate closed pores in coal-derived hard carbon. The reason for the formation of closed pores is that the uniform pores on the coal surface generated by the wear and etching of Zn_2_(OH)_2_CO_3_ are repaired at high temperatures. *Via* mechanism characterization, we verified that the plateau capacity is related to the filling of sodium ions in closed pores. Therefore, the as-prepared coal-derived hard carbon delivers a high capacity of 325.3 mA h g^−1^ (plateau capacity accounting for 45.1%) at a current density of 0.03 A g^−1^ with a capacity retention rate of 83.5% over 500 cycles. This work has demonstrated that reasonable pore design is an effective strategy to improve the electrochemical sodium storage performance of coal-derived hard carbon, providing an effective approach for the high value-added utilization of coal.

## Abstract 2

We propose a method of Zn_2_(OH)_2_CO_3_ assisted ball milling combined with subsequent carbonization to generate closed pores in coal derived hard carbon, leading to a high capacity of 325.3 mA h g^−1^ (plateau capacity accounting for 45.1%).

## Graphical abstract

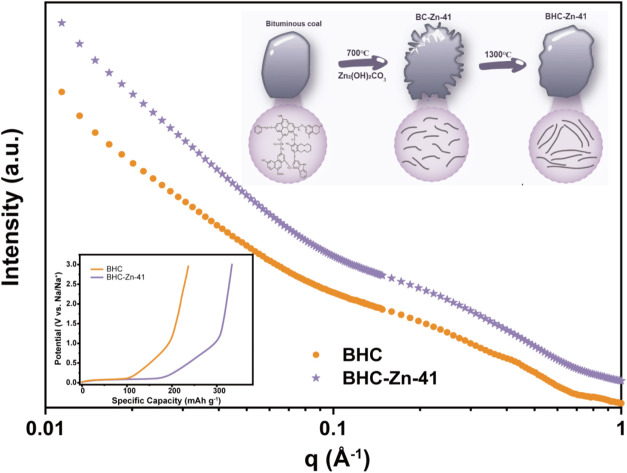

## Introduction

As a new generation of energy storage technology, sodium-ion batteries (SIBs) have attracted great attention for large-scale energy storage in the future.^1–3^ However, there is still a considerable challenge in terms of electrode materials based on the present status of development of SIBs, especially for anode materials.^4^ Conventional graphite cannot be used as the anode material for SIBs due to the limited performance, which is attributed to the thermodynamic instability of NaC_*x*_ and the small interlayer spacing of graphite.^5^ Therefore, researchers have been diligently looking for a suitable anode material.^6^

Hard carbon has received attention from researchers due to its low working potential and high theoretical specific capacity.^7–9^ Compared to other materials such as alloys, transition metal oxides, and organic electrodes, hard carbon was considered as the optimal choice as an anode for sodium-ion batteries because of its excellent physicochemical stability and electrochemical performance.^10–14^ The choice of precursors plays a key role in the cost and performance of hard carbon. Currently, biomass materials such as shaddock peel,^15^ corn straw,^16^*etc.,* are generally used as raw materials in mature hard carbon preparation processes*.* The major advantage of biomass-derived hard carbon is its high specific capacity. Nevertheless, just like polymer precursors, biomass-derived hard carbon suffers from low carbon yield which leads to high cost of preparation.

Coal, a solid combustible mineral, is formed from plant remains that have been buried underground and transformed through a long and complex process involving biochemistry, geochemistry and physicochemical changes.^17–19^ Disappointingly, the electrochemical performance of coal-derived hard carbon is poor. Structurally, coal has a higher inertinite composition, which leads to high carbon content and high yield after carbonization. However, due to the dense carbon structure, it is difficult to form a significant number of closed pores inside. According to the study of the sodium storage mechanism over the years, the plateau capacity is closely related to the number of closed pores.^20–22^ The filling of the closed pores with sodium ions (which may be accompanied by the formation of sodium clusters within the closed pores) will show a plateau capacity in the electrochemical charge/discharge profile. However, for coal, designing such closed pores is a rather difficult task because many conventional pore forming methods are not suitable for coal. For example, raw materials characterized by encapsulating the precursor with a hard template can form hard carbon rich in closed pores during carbonization and washing, but the hard template has difficulty entering the coal interior. In addition, some corrosive gases (such as CO_2_ and ethanol vapor) have difficulty flowing smoothly inside coal. Therefore, pore design requires more consideration for the structural characteristics of coal.

Herein, we applied a Zn_2_(OH)_2_CO_3_ assisted ball milling method to reduce the size of coal particles and to produce a uniform open pore structure by surface abrasion and etching. Then, the open pores were repaired in combination with subsequent high temperature carbonization to prepare coal-derived hard carbon materials with abundant closed pore structures. The structural characterization results indicate that there are a large number of uniformly sized closed pores in the coal derived hard carbon, leading to an improved specific capacity of 325.3 mA h g^−1^ at 0.03 A g^−1^ (146.7 mA h g^−1^ for plateau capacity). This strategy demonstrates the correlation between the closed pores and the plateau capacity. This work provides an effective and feasible strategy for the production of highly cost-effective coal-derived hard carbon materials.

## Results and discussion

**Fig. 1:**
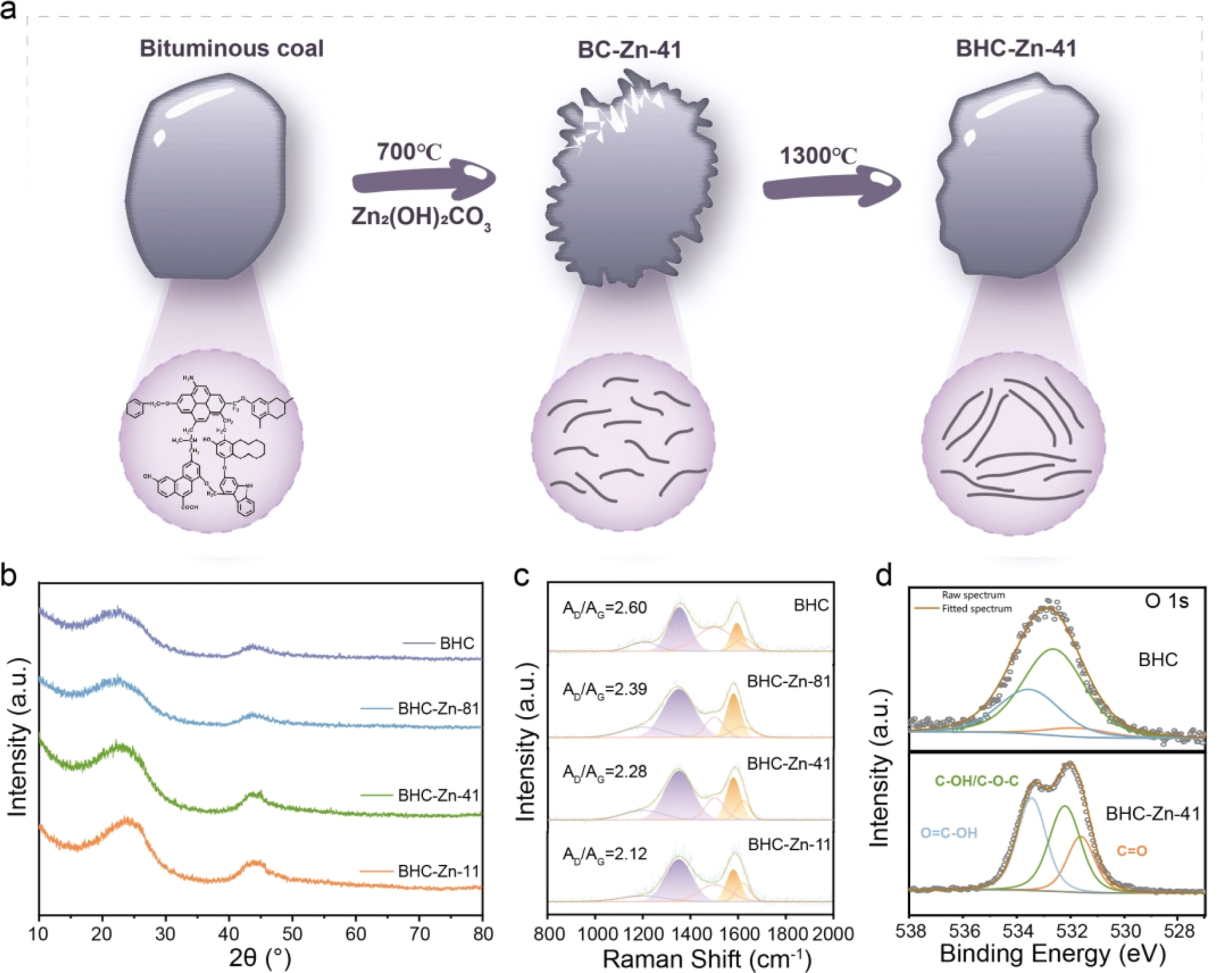
(a) Schematic representation of the preparation process for BHC-Zn; (b) XRD patterns and (c) Raman spectra of all samples; (d) high-resolution O 1s XPS spectra of BHC and BHC-Zn-41.

Fig. 1a presents the schematic illustration of the preparation of BHC-Zn-41. After ball milling with Zn_2_(OH)_2_CO_3_, annealing, and washing, the surface of coal particles is abraded and etched. The generated defects are repaired at high temperatures to form a closed pore structure. In order to understand the structural characteristics of coal-derived hard carbon anode materials, a series of samples were first characterized by XRD. The test results are shown in Fig. 1b; all four samples exhibit two broad peaks at around 24° and 43°, corresponding to the (002) crystal plane and (100) crystal plane of the hard carbon material, respectively.^23^ The position of the (002) peak can reflect the degree of graphitization of hard carbon materials.^24^ Furthermore, it can be clearly observed that after treatment with Zn_2_(OH)_2_CO_3_, the (002) peak of the sample gradually shifts towards a higher angle. The degree of shift is positively correlated with the proportion of Zn_2_(OH)_2_CO_3_. As the proportion of Zn_2_(OH)_2_CO_3_ increases, the (002) peak shifts more significantly and the peak intensity increases. The *d*_002_ interlayer spacing of the sample can be calculated based on the Bragg equation.^25^ The peak position values of BHC, BHC-Zn-81, BHC-Zn-41, and BHC-Zn-11 are 22.383, 22.708, 23.466, and 25.090, respectively. After calculation, the *d*_002_ interlayer spacings of samples BHC, BHC-Zn-81, BHC-Zn-41, and BHC-Zn-11 were 0.397, 0.391, 0.379, and 0.355 nm, respectively. The above results indicate that as the proportion of Zn_2_(OH)_2_CO_3_ increases, the degree of graphitization of hard carbon also increases. This may be due to zinc playing a catalytic role in graphitization.^26^ An appropriate increase in graphitization degree is conducive to the interlayer intercalation of sodium ions. The FWHMs of BHC, BHC-Zn-81, BHC-Zn-41, and BHC-Zn-11 are 7.167, 5.045, 3.728, and 2.430, respectively. The grain sizes calculated using the Scherrer formula are 1.120, 1.588, 2.151, and 3.311, respectively.

Fig. 1c shows the Raman spectra of the samples. The two broad peaks can be deconvoluted into four Lorentzian peaks, where the D band (1359 cm^−1^) is attributed to the A_1g_ vibration of the carbon rings derived from defects, and the G band (1600 cm^−1^) is assigned to the E_2g_ vibration of the sp^2^ carbon atoms.^27–29^ The integration area ratio between the D peak and the G peak (*A*_D_/*A*_G_) can reflect the degree of defects in the sample. Obviously, compared to BHC samples, the *A*_D_/*A*_G_ value of BHC-Zn is smaller, indicating that the pores and defects generated by Zn_2_(OH)_2_CO_3_ assisted ball milling are more easily repaired at high temperatures. During Zn_2_(OH)_2_CO_3_ etching, open pores are first formed on the carbon matrix, which are then reorganized to closed pores through high-temperature carbonization. After rearrangement, the carbon becomes more ordered, thereby reducing defects such as edges and heteroatoms.^30^ Therefore, the *A*_D_/*A*_G_ value of BHC-Zn-41 is smaller than that of BHC. In order to investigate whether Zn_2_(OH)_2_CO_3_ affects the element content and functional group types in coal-derived hard carbon during the decomposition process, XPS analysis was performed on all samples. The survey XPS spectrum is shown in Fig. S1,† each sample exhibits two peaks at approximately 293 eV and 532 eV, corresponding to the C 1s peak and O 1s peak, respectively, proving that the main components of the sample are carbon and oxygen elements.^31^ The content of the O element in sample BHC-Zn-41 is 11.62 at% compared to that in BHC (4.64 at%), indicating the formation of oxygen-containing functional groups in the sample BHC-Zn-41. The high-resolution O 1s XPS spectrum is presented in Fig. 1d and S2,† which displays three peaks located at 531.5, 532.7, and 533.3 eV, belonging to C=O, C–OH/C–O–C, and O=C–OH, along with chemically adsorbed oxygen functional groups, respectively.^32–34^ The area ratios of C=O peaks in samples BHC, BHC-Zn-81, BHC-Zn-41, and BHC-Zn-11 were 6.36%, 14.3%, 22.21%, and 10.05%, respectively. Among them, the C=O peak area ratio in BHC-Zn-41 is significantly higher than that in BHC, which may be attributed to the reaction between carbon and zinc oxide (formed during the decomposition process of Zn_2_(OH)_2_CO_3_): ZnO + C → Zn + C=O. The C=O can promote reversible sodium ion adsorption capacity, which is beneficial for improving the sodium storage capacity of coal-derived hard carbon anodes. The high-resolution C 1s XPS spectrum is shown in Fig. S3.† The peaks located near 284.7, 285.6, 286.7, and 290.2 eV correspond to the C–C, C–OR, C–OH, and COOR functional groups, respectively. It can be clearly observed in the C 1s spectrum of BHC-Zn-41 (Fig. S3c†) that there is a clear peak near 290.2 eV, indicating the presence of a significant number of COOR functional groups. The above XPS results indicate that BHC-Zn-41 has the highest oxygen content and the highest C=O double bond content. The optimal ratio of bituminous coal to Zn_2_(OH)_2_CO_3_ is 4 : 1, which is beneficial for improving the reversible specific capacity of coal-derived hard carbon anodes.

**Fig. 2:**
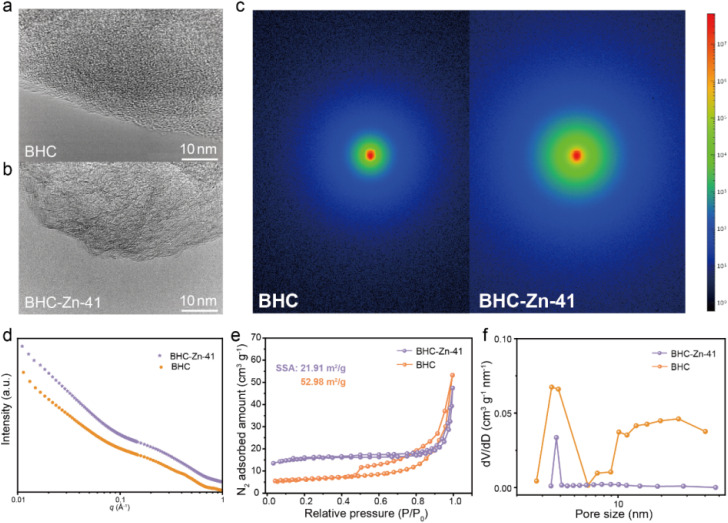
HRETM images of (a) BHC and (b) BHC-Zn-41; (c and d) SAXS patterns of BHC and BHC-Zn-41; (e) N_2_ adsorption/desorption isotherms and (f) pore size distribution of BHC and BHC-Zn-41.

The morphology and microcrystalline structure of the material were characterized *via* scanning electron microscopy (SEM) and high-resolution TEM (HRTEM). Fig. S4† shows the SEM images of BHC and BHC-Zn-41. It can be clearly observed that the particle size of the sample significantly decreases after treatment with Zn_2_(OH)_2_CO_3_. The reason for the small particle size of BHC-Zn-41 sample may be due to two factors: wear caused by Zn_2_(OH)_2_CO_3_ assisted ball milling, and etching of coal caused by gas generated during the decomposition of Zn_2_(OH)_2_CO_3_. A small particle size is beneficial for accelerating sodium ion transport. The HRTEM images of the four samples are shown in Fig. 2a and b and S5.† All four samples exhibit turbostratic structures, among which the microcrystalline structure of BHC is the most disordered with less obvious microcrystalline stripes.^35^ As the proportion of Zn_2_(OH)_2_CO_3_ increases, the microcrystalline structure gradually transitions from disordered to ordered, and the microcrystalline stripes gradually become longer and clearer, indicating an increase in the degree of graphitization of coal-derived hard carbon and an increase in the degree of internal structural order, which is consistent with the results of XRD and Raman. Closed pores are formed by the bending and folding of twisted graphene layers. As the proportion of Zn_2_(OH)_2_CO_3_ increases, the number of closed pores increases and becomes more pronounced, and the pore diameter increases (due to the elongation of graphene layers). In order to verify the closed pore structure of BHC-Zn-41, small angle X-ray scattering (SAXS) was used to detect closed micropores, as shown in Fig. 2c and d. The shoulder region within 0.1–0.4 Å^−1^ is attributed to scattering from nanopores.^36^ Obviously, the BHC-Zn-41 sample delivers higher scattering intensity than BHC, proving that the sample treated with Zn_2_(OH)_2_CO_3_ possesses more closed pores. The formation of these pores is caused by the *in situ* recombination of open pores during high-temperature carbonization.

The nitrogen adsorption and desorption isotherms of BHC and BHC-Zn-41 are shown in Fig. 2e; both samples exhibit typical Type IV isotherms, with a significant adsorption hysteresis loop in the medium pressure region (*P*/*P*_0_ = 0.3–0.8), indicating that their internal pore structure is mainly mesoporous.^37^ The nitrogen adsorption/desorption isotherm of BHC shows a large hysteresis loop area and a clear inflection point, indicating a large pore volume, complex pore structure, and poor connectivity in the material, mainly dominated by wedge-shaped pores. The hysteresis loop area of sample BHC-Zn-41 is relatively small, the inflection point is smoother, and the adsorption and desorption isotherms almost overlap, indicating that the detectable pore volume in this material is smaller and the connectivity is better than that of BHC. BHC-Zn-41 has a smaller SSA of 21.91 m^2^ g^−1^, while BHC has a larger SSA of 52.98 m^2^ g^−1^. After pretreatment with Zn_2_(OH)_2_CO_3_, residual zinc and its composites in the coal are removed by acid washing. During the subsequent carbonization at 1300 °C, there will not be a large amount of zinc and its composites remaining inside the sample. Carbon undergoes rearrangement at high temperatures, resulting in a smaller specific surface area. A smaller specific surface area is beneficial for reducing electrolyte decomposition and improving the initial coulombic efficiency (ICE) of sodium storage.^38^ According to the NLDFT method, the pore size distribution of the two samples was obtained, as shown in Fig. 2f. The pore size distribution in the BHC sample is uneven, mainly consisting of mesopores with a pore size of about 3.5 nm. The pores of BHC-Zn-41 are concentrated at approximately 3.7 nm, exhibiting a uniform pore size structure. This is because during the preparation process, bituminous coal is first mixed with Zn_2_(OH)_2_CO_3_*via* ball milling. During this treatment process, the particle size of bituminous coal decreases. During pre-carbonization at 700 °C, alkaline Zn_2_(OH)_2_CO_3_ decomposes to generate CO_2_ and ZnO. ZnO acts as a hard template to regulate the pore structure in the carbonization process of bituminous coal. Finally, after high-temperature carbonization, a coal-derived hard carbon with a uniform pore size is obtained.

**Fig. 3:**
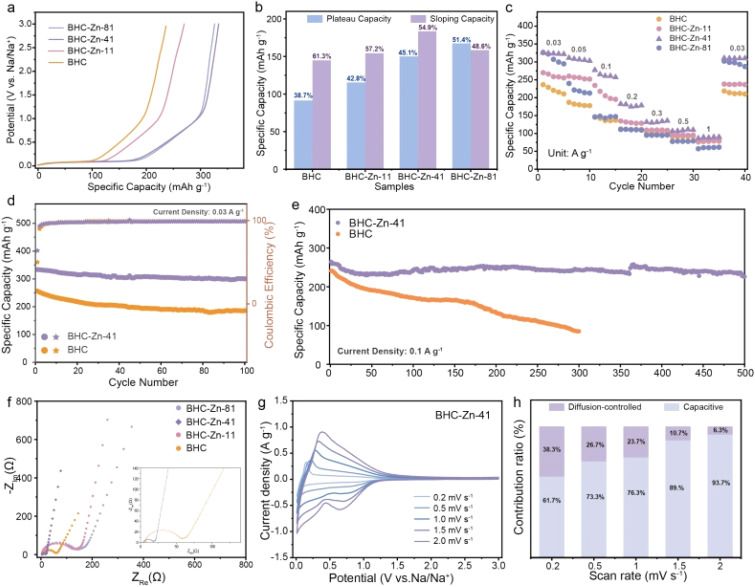
(a) Galvanostatic charge profiles of all four samples at 0.03 A g^−1^. (b) The percentage of capacity contribution of all four samples. (c) Rate performance of all four samples. Cycle performance of BHC and BHC-Zn-41 at (d) 0.03 A g^−1^ and (e) 0.1 A g^−1^. (f) Nyquist curves of all four samples. (g) CV curves of BHC-Zn-41 at various scan rates. (h) Comparison of contribution ratios of capacitive process and diffusion-controlled at various scan rates.

Electrochemical testing was used to verify the effect of structural changes on sodium storage performance. Fig. 3a shows the initial cycle charging curves of the four samples. It can be observed that all four samples exhibit similar charging curves, with a plateau area below 0.1 V and a slope area above 0.1 V. BHC-Zn-41 exhibits the highest charging specific capacity, with a capacity of 325.3 mA h g^−1^ at a current density of 0.03 A g^−1^. BHC-Zn-41 was compared with other recently related coal based carbon materials in Table S1,† demonstrating its excellent capacity. Fig. 3b compares and analyzes the capacities of the slope and plateau regions in the charging curves of different materials. It is evident that after treatment with Zn_2_(OH)_2_CO_3_, the plateau capacity and proportion of prepared hard carbon are significantly increased, which can be attributed to the improvement of interlayer order and the increase of closed pores inside the hard carbon. As the proportion of Zn_2_(OH)_2_CO_3_ increases, the plateau capacity and proportion in the total capacity gradually decrease. This may be due to the increase in the degree of internal order of hard carbon, the gradual decrease in interlayer spacing, and the thicker walls of closed pores formed by bent graphene layers, which are not conducive to the insertion and extraction of sodium ions in the interlayer and closed pores.

The rate performance of the four samples is shown in Fig. 3c. The charge–discharge curves of sample BHC-Zn-41 at different current densities are shown in Fig. S6.† It can be observed that as the current density increases, the plateau capacity and slope capacity in the charge–discharge curves of BHC-Zn-41 gradually decrease. At all current densities, the specific capacity of BHC-Zn-41 is higher than that of other samples. The reversible specific capacities of 311.1, 277.1, and 182.3 mA h g^−1^ were achieved at current densities of 0.05, 0.1, and 0.2 A g^−1^, respectively. Subsequently, cycle stability tests were conducted on sample BHC and BHC-Zn-41, as shown in Fig. 3d. After 100 cycles at a current density of 0.03 A g^−1^, the reversible specific capacity can still remain at 300.4 mA h g^−1^, and the capacity retention rate can reach 90.2%, which is superior to that of the BHC sample. In addition, performance was also tested at a high current density of 0.1 A g^−1^. After 500 cycles, the reversible specific capacity still remain at 226.1 mA h g^−1^ with a capacity retention rate of 83.5% (Fig. 3e). The capacity of BHC continues to decrease during the cycling process, and the reversible specific capacity is only 84.9 mA h g^−1^ after 300 cycles. This is because BHC-Zn-41 has a higher degree of graphitization compared to BHC, which is conducive to rapid ion transport. Moreover, it has more closed pores and sodium storage sites, which are conducive to sodium storage, and thus exhibits higher reversible specific capacity and excellent cycling stability.^39^

The resistance characteristics of the samples were characterized through AC impedance testing, and the test results are shown in Fig. 3f. From the graph, it can be seen that the semicircle in the Nyquist plot of sample BHC-Zn-41 is the smallest, and the slope of the low frequency region curve is the largest.^40^ In addition, the EIS test results were fitted using an equivalent circuit model (Fig. S7†), and the fitting results are shown in Table S2.† Sample BHC-Zn-41 delivers the smallest *R*_ct_ and *R*_Ω_ values, which are 12.07 Ω and 4.23 Ω, respectively. The above results indicate that the charge transfer impedance is the smallest and the ion diffusion is the fastest in sample BHC-Zn-41.^41^ Therefore, sodium ions can be quickly transferred under high current, exhibiting excellent rate performance. Fig. S8† shows the CV curves of the four samples at a scanning rate of 0.1 mV s^−1^. The initial CV curves of each sample are similar, showing a pair of sharp oxidation–reduction peaks at approximately 0.1 V, corresponding to the plateau region in the GCD profiles. The oxidation–reduction peak of sample BHC-Zn-41 is sharpest at 0.1 V, with the highest peak current density, indicating that it has the fastest reaction kinetics. An irreversible reduction peak was observed at approximately 0.5 V, mainly caused by the decomposition of the electrolyte and the formation of the SEI film, corresponding to the irreversible capacity during the first charge–discharge process.^42^ It can be clearly observed from the four samples that the area of the irreversible region in the CV curve of sample BHC-Zn-41 is the smallest, indicating that its irreversible capacity is the lowest. In the subsequent cycles, the CV curves of BHC-Zn-41 highly overlap, indicating that the reaction is highly reversible. In order to gain a deeper understanding of the sodium storage kinetics of the optimal sample BHC-Zn-41, CV tests were conducted at different scanning rates. Fig. 3g shows the CV profiles of BHC-Zn-41 at different scanning rates. It can be observed that as the scanning rate increases, there is little difference in the variation of the CV profile, demonstrating excellent dynamic characteristics.^43^ The capacitive contribution and diffusion-controlled contribution of this material at different scanning rates were calculated *via* Dunn's method.^44^ Fig. S9† shows the capacitive contribution (purple shaded area) of BHC-Zn-41 at 0.5 mV s^−1^, accounting for 73%. At the same time, the contribution of capacitance and diffusion at other scanning rates were also calculated (Fig. 3h). It can be observed that as the scanning rate increases, the contribution of the capacitive process gradually increases (61.7% to 93.7%), while the diffusion-controlled contribution gradually decreases (38.3% to 6.3%). The reason for the decrease in the proportion of the diffusion process is that sodium ions are difficult to enter the interior of hard carbon at higher scanning rates. The higher contribution of capacitance indicates that BHC-Zn-41 has faster ion transport, thus exhibiting excellent performance at high current densities, corresponding to its excellent rate performance.

**Fig. 4:**
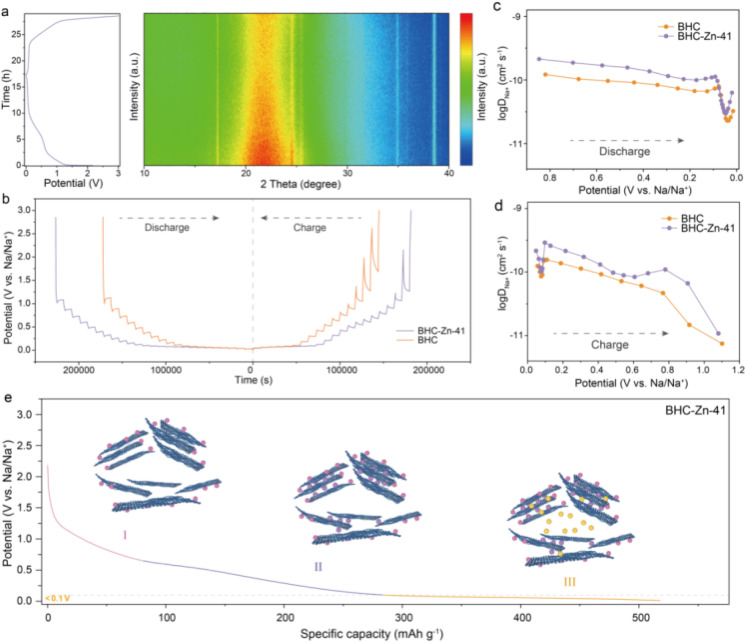
(a) *In situ* XRD spectra of BHC-Zn-41 during the first discharge–charge process at 0.03 A g^−1^. (b) GITT potential profiles and (c and d) the calculated Na^+^ diffusion coefficients during the charge/discharge process. (e) Illustration of the sodium storage mechanism.

*In situ* XRD analyses were performed to investigate the sodium ion storage mechanism in the BHC-Zn-41 electrode. As shown in Fig. 4a, the potential *versus* time curves and *in situ* XRD patterns of the BHC-Zn-41 electrode during the first charging and discharging process at 0.03 A g^−1^ are demonstrated. It can be observed that during the discharging processes, the position of the (002) peak shifts towards a smaller angle, and the peak intensity decreases, proving the intercalation of sodium ions. The diffusion characteristics of sodium ions in materials were measured *via* the galvanostatic intermittent titration technique (GITT).^45^Fig. 4b shows the GITT curves of samples BHC and BHC-Zn-41 during the charge and discharge processes. The diffusion coefficient *D*_Na^+^_ of sodium ions in the two samples was calculated as a function of potential, as shown in Fig. 4c and d. Clearly, the curves of the two samples exhibit similar trends of change. At the beginning of discharge, sodium ions first adsorb on the surface and defects of hard carbon materials.^46^ The adsorption process occurs quickly, so the *D*_Na^+^_ value is relatively high. As the potential decreases, sodium ions are inserted between the layers of hard carbon materials, resulting in a decrease in *D*_Na^+^_ value. As the discharge reaches 0.1 V, sodium ions begin to fill the pores, resulting in an increase in *D*_Na^+^_. There are abundant sodium storage sites and unique large and uniform closed pores in BHC-Zn-41. In addition, BHC-Zn-41 also delivers more ordered carbon layer structures, which is conducive to the insertion and extraction of sodium ions. Therefore, the *D*_Na^+^_ value of sample BHC-Zn-41 is always greater than that of BHC. The sodium storage mechanism underlying HC can be succinctly delineated in Fig. 4e. During the discharge process, sodium ions first adsorb onto the surface and defects of hard carbon. As the voltage decreases, sodium ions begin to intercalate into the carbon layer and exhibit a sloping capacity. After discharging to around 0.1 V, sodium ions begin to fill the closed pores and generate high plateau capacity.

**Fig. 5:**
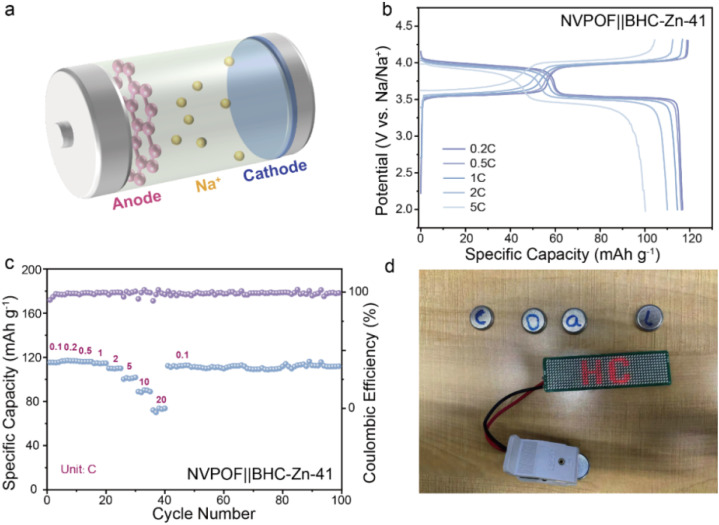
(a) Schematic of the sodium-ion full-cell with the BHC-Zn-41 anode and NVPOF cathode. (b) The GCD curves at various current densities and (c) rate performance of the BHC-Zn-41‖NVPOF full cell. (d) A photo shows that a BHC-Zn-41‖NVPOF full-cell can light up 52 LED bulbs.

In order to evaluate the possibility of the coal-derived hard carbon anode material BHC-Zn-41 being applied in practical production, a full cell was matched with Na_3_V_2_O_2_(PO_4_)_2_F (NVPOF) as the cathode for electrochemical performance testing. Pre-sodiation was carried out on the negative side by the contact method prior to assembly of the full cell, with a mass ratio of cathode and anode active materials of approximately 1.1 : 1. Fig. 5a shows a simple schematic of the full cell, with pink representing BHC-Zn-41 on the anode side, blue representing NVPOF on the cathode side, and the yellow particles representing the free-moving sodium ions. The electrochemical performance of the full-cell NVPOF‖BHC-Zn-41 at different current densities is shown in Fig. 5b and c. At a current density of 0.2C (1C = 130 mA g^−1^), the reversible specific capacity is 116.8 mA h g^−1^ and the energy density is as high as 436.7 W h kg^−1^ (based on the cathode mass). In addition, the full cell also demonstrated excellent rate performance and cycling stability, with a capacity of up to 94.3 mA h g^−1^ at 10C (about 79.9% of that at 0.1C) and 112.7 mA h g^−1^ after 100 cycles at 0.1C (with capacity retention of up to 98.6%). Fig. 5d demonstrates that the assembled full cell can power 52 light emitting diode (LED) bulbs in series. The excellent electrochemical performance demonstrates that the material is promising for mass production applications as a commercial anode for sodium-ion batteries.

## Conclusions

Coal-derived hard carbon rich in closed pores (BHC-Zn-41) is prepared *via* a Zn_2_(OH)_2_CO_3_ assisted ball milling method followed by carbonization. Compared to the samples prepared by direct annealing, BHC-Zn-41 has a smaller particle size, higher oxygen content, and more closed pores. Therefore, the BHC-Zn-41 sample delivers better capacity and diffusion properties with a capacity of 325.3 mA h g^−1^ at a current density of 0.03 A g^−1^. In addition, combined with mechanism analysis, the correlation between closed pores and plateau capacity has been proven. This work provides profound guidance for the structural design of future hard carbon materials. However, coal derived hard carbon still has a high specific surface area, which is not conducive to the energy of the full cell. Therefore, in the future, the preparation of coal derived hard carbon with a low specific surface area is an important development trend, which can be achieved by combining other carbons.

## Experimental

### Material synthesis

The bituminous coal used in this work was sourced from Yulin City, Shaanxi Province, China. After the bituminous coal was crushed into powder by using a crusher, it was washed with 5 M HCl and 6 M NaOH at 60 °C for 12 hours, then washed with deionized water and dried. Next, the obtained coal powder and Zn_2_(OH)_2_CO_3_ were mixed evenly in a planetary ball mill in different mass ratios (500 rpm, 8 h). The mixed powder was annealed in a tube furnace under an argon atmosphere at 700 °C for 2 hours, and residual zinc compounds were removed with 1 M HCl solution. The dried black powder was carbonized in a tube furnace for 2 hours in an argon atmosphere at 1300 °C to obtain a series of coal-derived hard carbon materials. The products obtained with the mass ratios of 1 : 1, 4 : 1, and 8 : 1 for bituminous coal and Zn_2_(OH)_2_CO_3_ are labeled as BHC-Zn-11, BHC-Zn-41, and BHC-Zn-81, respectively. In addition, the coal-derived hard carbon material obtained by direct high-temperature carbonization of bituminous coal after impurity removal at 1300 °C for 2 hours is labeled as BHC.

### Materials characterization

X-ray diffraction (XRD) analysis was conducted using a Rigaku Smart Lab X-ray diffractometer with settings of 30 mA, 40 kV, and *λ* = 1.5418 Å. TEM images were obtained with a JEM-2100F TEM (JEOL, Japan). The LabRAM HR Evolution Raman spectrometer (Horiba) was used to measure the Raman spectra. X-ray photoelectron spectra (XPS) were collected using a VG Scientific apparatus with a 300 W Al Kα source. The specific surface areas and pore-size distributions of the products were estimated using a Micromeritics ASAP 2020 analyzer. The surface area was calculated using the Brunauer–Emmett–Teller (BET) equation, and the pore size distribution was analyzed with non-local density functional theory (NLDFT).

### Electrochemical measurements

The electrochemical performances of carbon materials were characterized in 2032-type half cells. The carbon materials, conductive agent super C45 and PVDF with a weight ratio of 7 : 2 : 1 are mixed in *N*-methyl-2-pyrrolidinone (NMP). The slurry was pasted on Al foil and fully dried at 100 °C for 24 h under vacuum. The round disks with a diameter of 12 mm were used as working electrodes. Sodium metal foil, glass fibers and 1 M NaPF_6_ in EC : DEC (v/v 1 : 1) were applied as the counter electrode, separator and electrolyte, respectively. All the coin cells were assembled in an Ar glovebox. The galvanostatic charge/discharge properties were evaluated on a LAND CT 2001A battery testing system (CT2001A, Wuhan LAND Corporation, China). CV curves were obtained from the electrochemical workstation CHI 660E (Shanghai Chenhua, China) between 0.001 and 3 V. The GITT test of the half-cell was conducted using the Arbin Instrument, with a pulse current of 0.03 A g^−1^, a charge–discharge time of 0.5 h, and a relaxation time of 2 h. The *in situ* XRD analyses were carried out using a special *in situ* cell with a beryllium window for X-ray penetration. The *in situ* cell was carried by GCD measurement at a current density of 0.05 A g^−1^ between 0.001 and −3 V. In the assembly of the full cell, the pre-sodiation of the negative electrode was carried out by directly contacting the negative electrode with metallic sodium covered with electrolyte for 2 hours.

## Data availability

The data supporting this article have been included as part of the ESI† and in the main text.

## Author contributions

Wen-Yu Qian and Xin-Yang Zhou: methodology, data curation, writing – original draft; Xin-Yao Liu: formal analysis, investigation, software; Meng-Yuan Su and Kai-Yang Zhang: validation, visualization; Xing-Long Wu: conceptualization, funding acquisition, supervision, writing – review and editing.

## Conflicts of interest

There are no conflicts to declare.

## Supplementary Material

SC-OLF-D4SC06549B-s001
