# Supplementary material for: Breakage of the dense structure of coal precursors increases the plateau capacity of hard carbon for sodium storage
Source: Chem Sci. 2024 Dec 2;16(1):104–12. doi: 10.1039/d4sc06549b (PMC11609975; doi:10.1039/d4sc06549b)
Supplement: SC-016-D4SC06549B-s001 [file SC-016-D4SC06549B-s001.pdf]

Supplementary Information (SI) for Chemical Science.
This journal is © The Royal Society of Chemistry 2024

Breakage of dense structure of coal precursor increases plateau capacity of hard carbon for sodium storage

Wen-Yu Qian,[a,b] Xin-Yang Zhou,[a] Xin-Yao Liu,[b] Meng-Yuan Su,[b] Kai-Yang Zhang,[a] Xing-Long Wu*[a,b]

[a] MOE Key Laboratory for UV Light-Emitting Materials and Technology, Northeast Normal University, Changchun, Jilin 130024, China, Email: xinglong@nenu.edu.cn (X.-L. Wu).

[b] Faculty of Chemistry, Northeast Normal University, Changchun, Jilin 130024, China

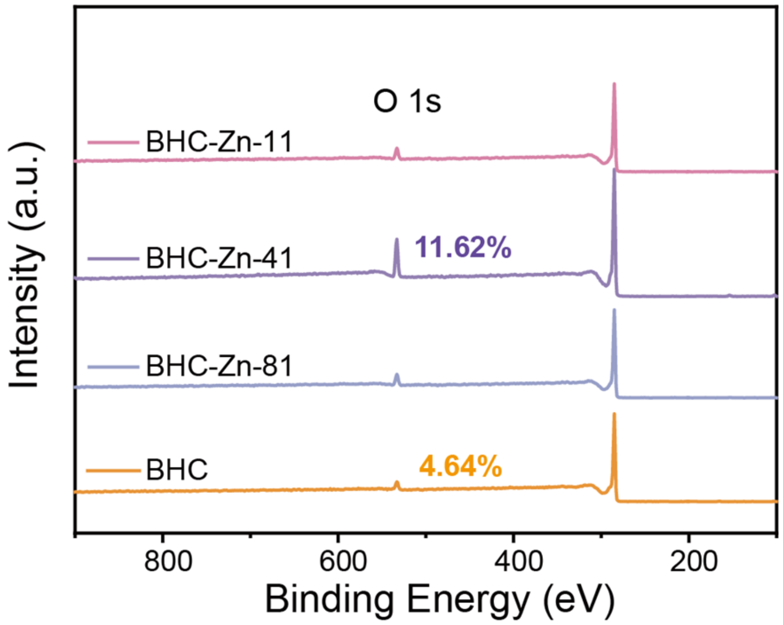

Fig. S1 XPS survey spectra of BHC and BHC-Zn.

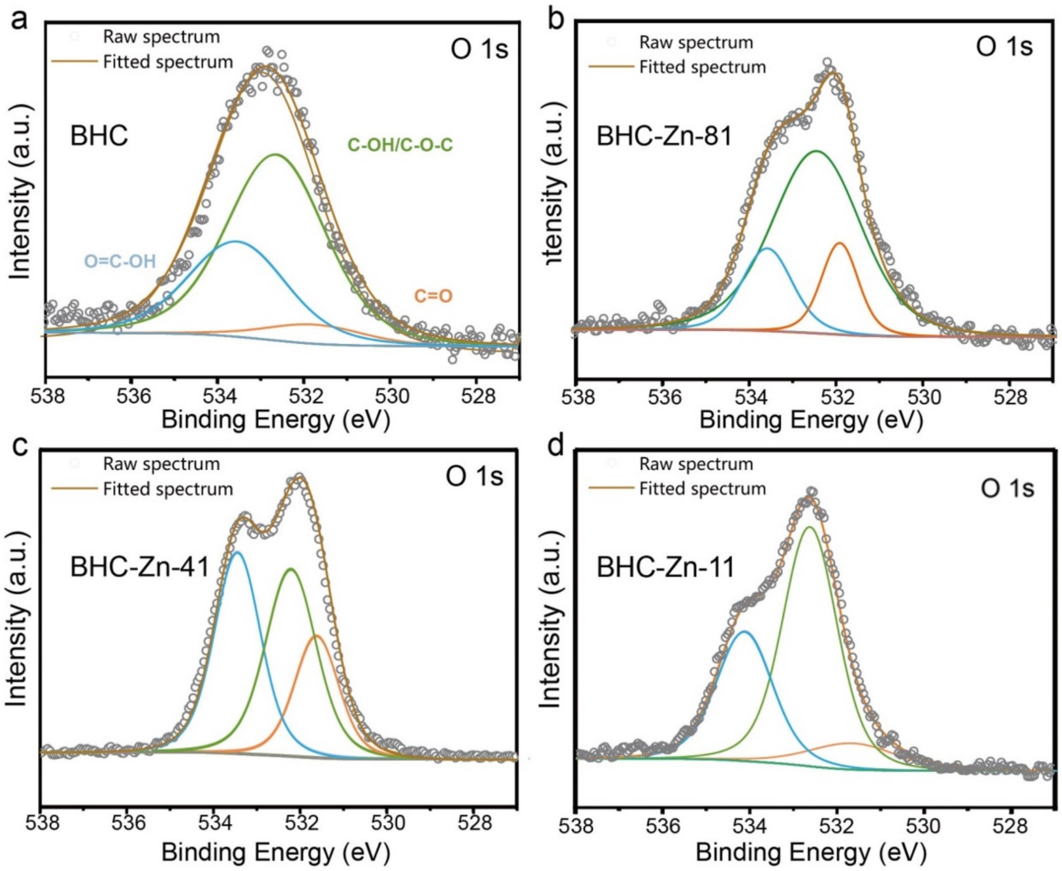

Fig. S2 High-resolution O 1s spectra of BHC and BHC-Zn.

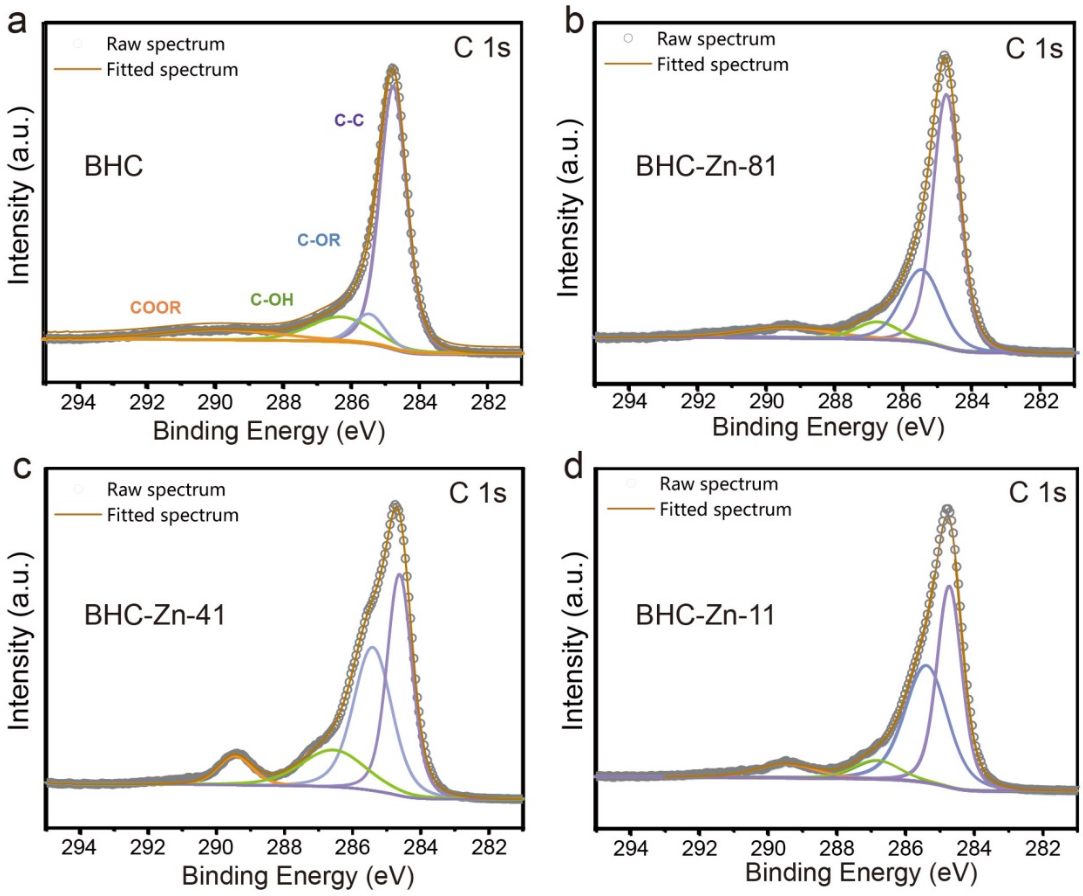

Fig. S3 High-resolution C 1s spectra of BHC and BHC-Zn.

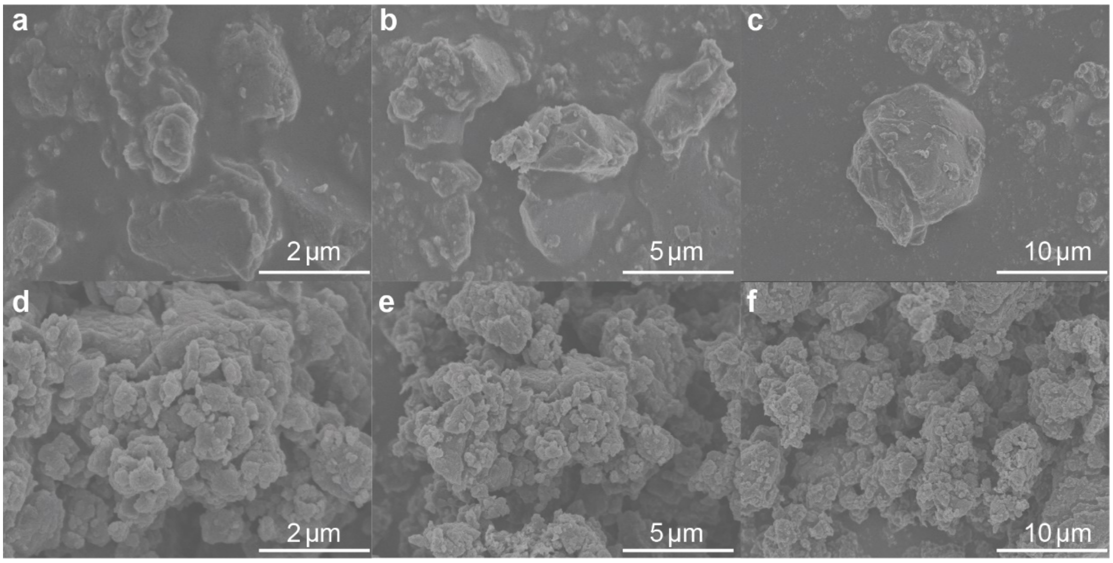

Fig. S4 SEM of (a, b, c)BHC and (d, e, f)BHC-Zn-41.

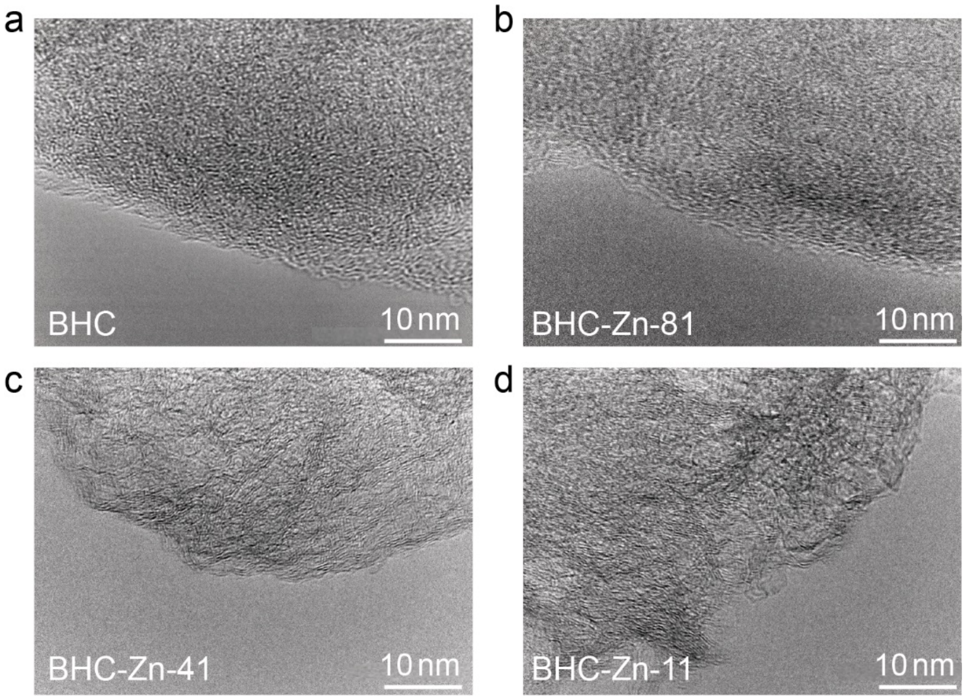

Fig. S5 HRTEM of (a)BHC, (b)BHC-Zn-11, (c)BHC-Zn-41 and (d)BHC-Zn-81.

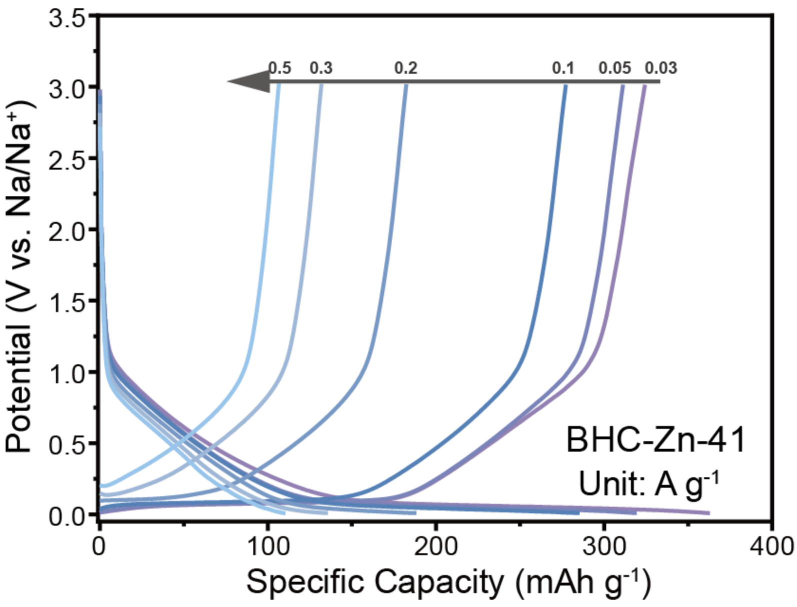

Fig. S6 The charge discharge curves of sample BHC-Zn-41 at different current densities.

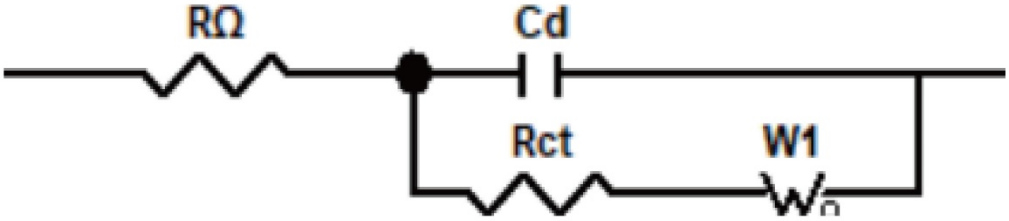

Fig. S7 equivalent circuit fitting diagram

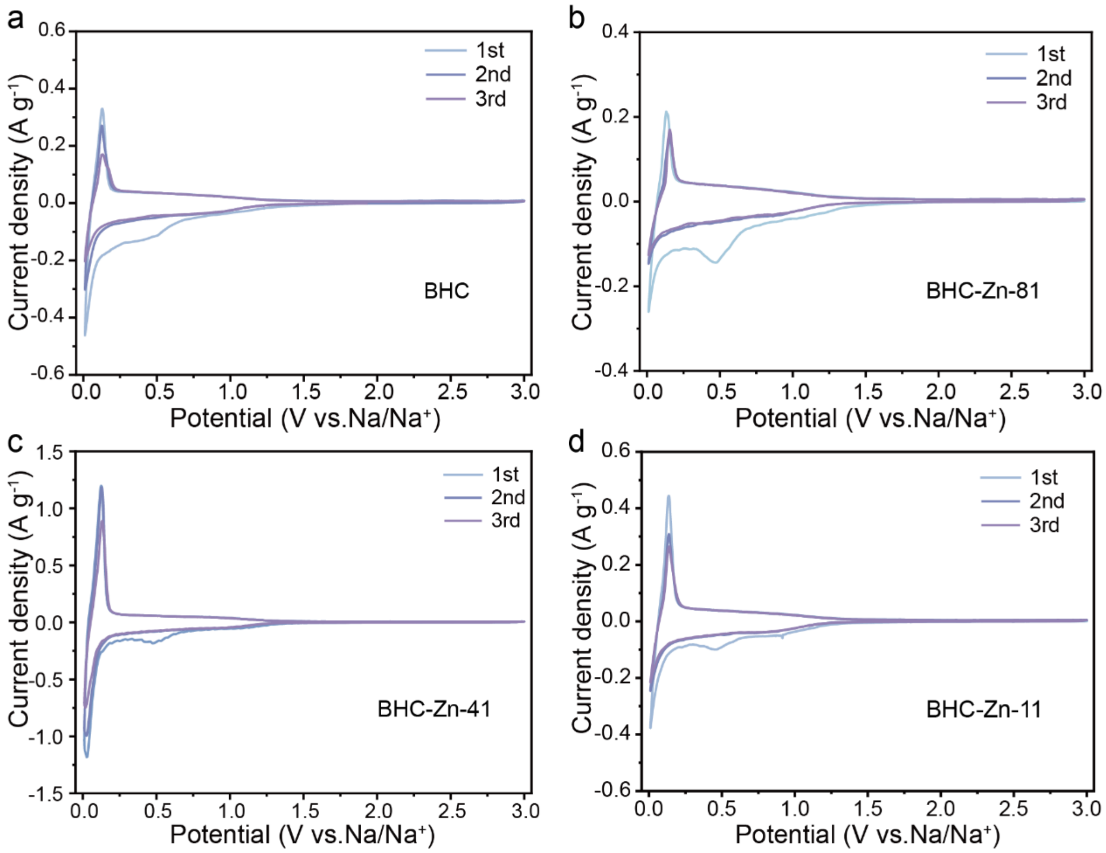

Fig. S8 the CV curves of (a)BHC, (b)BHC-Zn-11, (c)BHC-Zn-41 and (d)BHC-Zn-81 at 0.1 mV s^{-1}

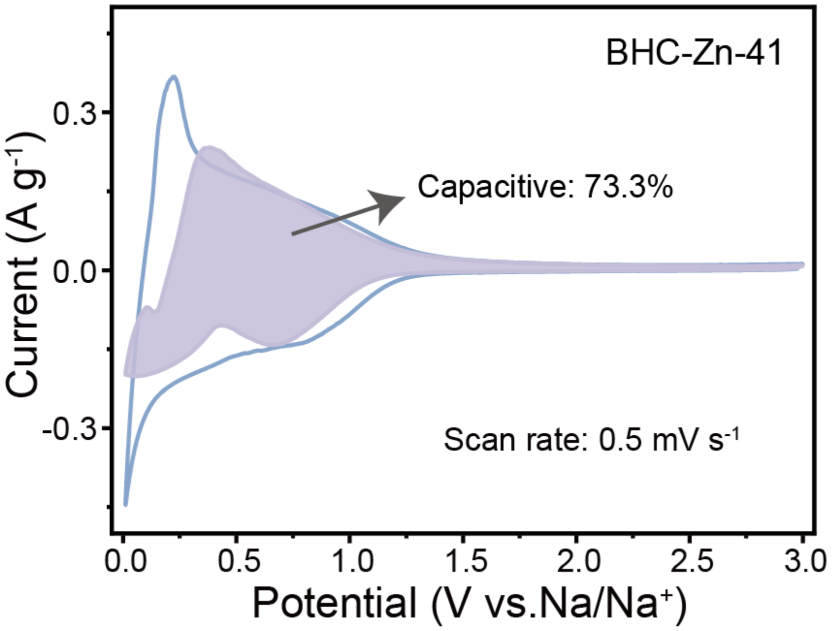

Fig. S9 CV curve of BHC-Zn-41 at the scan rate of 0.5mV s^{-1}. Shaded region exhibits the capacitive contribution.

Table S1 A literature comparison with capacity for coal derived carbon

Sample	Capacity ($mAh\ g^{-1}$)	Current Density	Reference
**Coal-based hard carbon with NH_3 treatment**	220	0.1 A g^{-1}	[1]
**Porous flaky HC derived from coal**	303.6	50 mA g^{-1}	[2]
**Coal based hard carbon with fast carbonization**	292	0.1 C	[3]
**Bituminous coal derived carbon**	287.1	20 mA g^{-1}	[4]
**Coal derived hard carbon**	306.3	30 mA g^{-1}	[5]
**BHC-Zn-41**	325.3	30 mA g^{-1}	This work

Table S2 Equivalent circuit fitting results of BHC and BHC-Zn.

Samples	R_{Ω} (+)	R_{ct} (+)
**BHC-Zn-11**	4.09	108.31
**BHC-Zn-41**	4.23	12.07
**BHC-Zn-81**	3.82	118.12
**BHC**	6.87	44.25

Reference

[1] R. Li, B. Yang, A. Hu, B. Zhou, M. Liu, L. Yang, Z. Yan, Y. Fan, Y. Pan, J. Chen, T. Lia , K. Li, J. Liu, and J. Long, *Carbon*, **2023**, 215, 118489.

[2] X.-Y. Wang, K.-Y. Zhang, M.-Y. Su, H.-H. Liu, Z.-Y. Gu, D. Dai, B. Li, J.-W. Wang, X.-Y. He, and X.-L. Wu, *Carbon*, **2024**, 229, 119526.

[3] H. Wang, F. Sun, Y. Wang, D. Wu, J. Gao, J. Wang, and J. Gao, *Carbon*, 2024, 229, 119528.

[4] G. Liu, J. Yuan, H. Li, Z. Li, C. Hu, X. Qiao, M. Wang, B. Yuan, P. Zhang, and Z. Wu, *ACS Appl. Mater. Interfaces*, **2024**, 16, 46226−46236.

[5] M.-Y. Su, K.-Y. Zhang, Edison H. Ang, X.-L. Zhang, Y.-N. Liu, J.-L. Yang, Z.-Y. Gu, F. A. Butt, and X.-L. Wu, *Rare Met.*, **2024**, 43(6):2585–2596.
